# Radiation Biomarkers in Large Scale Human Health Effects Studies

**DOI:** 10.3390/jpm10040155

**Published:** 2020-10-03

**Authors:** Jayne Moquet, Kai Rothkamm, Stephen Barnard, Elizabeth Ainsbury

**Affiliations:** 1 Public Health England, Centre for Radiation, Chemical and Environmental Hazards, Chilton, Didcot, Oxfordshire OX11 0RQ, UK; k.rothkamm@uke.de (K.R.); stephen.barnard@phe.gov.uk (S.B.); liz.ainsbury@phe.gov.uk (E.A.); 2 Department of Radiotherapy & Radio-Oncology, University Medical Centre Hamburg-Eppendorf, 20251 Hamburg, Germany

**Keywords:** ionizing radiation, biomarkers, dicentric assay, micronucleus assay, gamma H2AX foci assay, health surveillance analyses

## Abstract

Following recent developments, the RENEB network (Running the European Network of biological dosimetry and physical retrospective dosimetry) is in an excellent position to carry out large scale molecular epidemiological studies of ionizing radiation effects, with validated expertise in the dicentric, fluorescent in situ hybridization (FISH)-translocation, micronucleus, premature chromosome condensation, gamma-H2AX foci and gene expression assays. Large scale human health effects studies present complex challenges such as the practical aspects of sample logistics, assay costs, effort, effect modifiers and quality control/assurance measures. At Public Health England, the dicentric, automated micronucleus and gamma-H2AX radiation-induced foci assays have been tested for use in a large health effects study. The results of the study and the experience gained in carrying out such a large scale investigation provide valuable information that could help minimise random and systematic errors in biomarker data sets for health surveillance analyses going forward.

## 1. Introduction

One of the hallmarks of ionizing radiation is its ability to induce DNA double-strand breaks, lesions that are difficult to repair and prone to mis-repair, resulting in mutations and chromosomal aberrations such as dicentrics or acentric fragments [1]. In addition, the modification or accumulation of DNA damage response proteins at the site of DNA double-strand breaks can be visualised as gamma-H2AX and 53BP1 foci [2]. These biomarkers of radiation exposure and effect are key tools to (i) support the clinical management of critically exposed individuals and reassure the ‘worried well’ following a radiation accident or incident, e.g., [3]; (ii) support counselling on the likelihood of long-term health radiation-induced effects such as cancer, e.g., [4]; (iii) provide dose estimates in epidemiological studies of health risks associated with radiation exposure, e.g., [5]; and (iv) predict the response of a tumour and the patient’s normal tissues to radiotherapy in order to personalise treatment and thus maximise the probability of complication-free tumour control, e.g., [6].

To date, DNA and chromosome damage-associated markers have been most widely used for biological dose estimation [7]. Considerable progress has been made recently in the development, standardisation and validation of exposure biomarkers, e.g., through the European Union (EU) projects MULTIBIODOSE (Multi-disciplinary biodosimetric tools to manage high scale radiological casualties; https://cordis.europa.eu/project/id/241536) and RENEB (Realizing the European Network of Biodosimetry; http://cordis.europa.eu/project/id/295513). Progress in the area of predictive markers of individual response to radiation exposure has been much more limited, although a number of small scale studies (7–10 patients plus matched controls) and recently larger studies (42–>1000 patients) have also suggested an association of DNA double-strand break-related markers or apoptosis with clinical radiosensitivity in non-syndromic patients [8,9,10,11,12,13].

The EU RENEB project has resulted in a number of key outputs and these include harmonisation activities to strengthen radiation exposure assessment, with a training program to include periodic inter-comparisons [14], a strategic research agenda [15] and a common quality manual outlining the framework of quality assurance/quality management (QA/QM), with QA/QM criteria for all assays that make up the operational basis of the network [16]. These assays include (i) the dicentric assay; (ii) the FISH-translocation assay; (iii) the micronucleus assay; (iv) the premature chromosome condensation assay; (v) the gamma-H2AX assay; and (vi) electron paramagnetic resonance/optically stimulated luminescence. In addition, candidate techniques, such as gene or micro-RNA expression, and new partner laboratories are being evaluated for inclusion in the network [17]. The network now has a legal structure with a minor adaptation in the name, RENEB—Running the European Network of biological dosimetry and physical retrospective dosimetry (https://www.reneb.net/). As of January 2016, RENEB has 26 member organisations and is now in a position not only to respond to radiation emergencies, but also to contribute to large scale studies relevant for patient health and radiation protection that require dose assessment [14].

Recent work at Public Health England (PHE) involved a large scale study of more than 400 historically exposed breast and prostate radiotherapy patients in which a panel of DNA damage-associated markers were used for biological dose reconstruction and as indicators of individual radiosensitivity following the ex vivo radiation exposure of blood samples. Based on this experience, discussed here are the complex challenges that need to be addressed when using such assays in large scale human health effects studies. These include practical aspects such as assay costs, effort, automation, sample logistics and failure rates, effect modifiers such as inter-scorer variation and assay ‘drift’ over time, but also quality control/assurance measures that could help minimise random and systematic errors in biomarker data sets.

## 2. Materials and Methods

### 2.1. Patient Selection and Blood Sampling

Volunteers participating in two randomised controlled radiotherapy trials, testing of 5.7 Gy and 6.0 Gy fractions of whole breast radiotherapy FAST or Conventional Hypofractionated High Dose Intensity Modulated Radiotherapy for Prostate Cancer (CHHIP), were recruited for the present study. FAST is a prospective randomised clinical trial testing 5.7 Gy and 6.0 Gy fractions of whole breast radiotherapy in terms of late normal tissue responses and tumour control (ISRCTN62488883) [18]. CHHIP (Conventional Hypofractionated High Dose Intensity Modulated Radiotherapy for Prostate Cancer (ICRCTN97182923)) is a randomised trial to see whether hypofractionated radiotherapy schedules for localised prostate cancer could improve the therapeutic ratio by either improving tumour control or reducing normal tissue side effects [19]. Both trials test the hypothesis that fewer, larger radiotherapy doses than those delivered by standard regimens improve local control and/or reduce late adverse effects in patients treated for early stage breast cancer (FAST) or localised prostate cancer (CHHIP). The trials collected prospective clinician and patient self-assessed scores of late adverse effects and quality of life for a minimum of 5 years post-treatment. The present study was carried out in accordance with the Declaration of Helsinki (1964) and the Research Governance Framework Second edition (2005). Ethics approval was obtained from all the participating cancer centres and written informed consent was given by all the study volunteers. Patient confidentiality was maintained at all times. Heparinized venous blood was collected from a total of 406 volunteers when they came in for one of their periodic post-treatment clinical assessments, over a period of 18 months, at selected UK centres participating in the FAST and CHHIP trials. Samples were packaged according to the UN 3373 Biological Substance Category B specifications [20] and dispatched by express courier, at ambient temperature, to PHE (10 mL per patient).

### 2.2. Ex Vivo Irradiation of Separated Lymphocytes and Whole Blood

Upon arrival at the laboratory the next day, blood was immediately aliquoted for use in the gamma-H2AX foci assay (FCA), the dicentric (DCA) and micronucleus (MNA) assays. Prior to irradiation, the whole blood for the gamma-H2AX assay was separated on histopaque 1077 (Sigma-Aldrich, Dorset, UK) and isolated lymphocytes suspended in minimal essential medium (MEM) with 10% heat-inactivated foetal bovine serum (FBS), 100 units/mL penicillin plus 100 µg/mL streptomycin and 2 mM L-glutamine (all from Invitrogen, Paisley, UK). Separated lymphocytes and whole blood samples were irradiated with X-rays using an A.G.O. X-ray System, model CP160/1 (AGO X-ray Ltd., Martock, UK), at 250 kVp with a half value layer of 2 mm of copper and 1.5 aluminium and a dose rate of 0.5 Gy/min. Irradiation of the samples was performed at 37 °C in 15 mL centrifuge tubes, held inside a 22 mm polystyrene block on top of an 8 mm Perspex plate. Dosimetry was carried out in the same geometry as the specimens using a calibrated reference ionisation chamber. All the exposures were monitored using a calibrated UNIDOSE electrometer, traceable to a national standard, and an ‘in beam’ ionisation chamber (all from PTW, Freiburg, Germany). In addition, the spatial dose uniformity was checked using Gafchromic EBT2 films (Vertec Scientific Ltd., Reading, UK). Separated lymphocytes for the FCA were irradiated to 0.5 and 4 Gy and the whole blood to 6 and 2 Gy for the DCA and MNA, respectively. Small scale studies, with a highly select group of patients, have suggested that the doses used here can predict normal tissue radiosensitivity using DCA [8], MNA [21] and FCA [9]. Sham-irradiated controls were also included for the FCA and DCA, but not for the MNA, due to the limited amount of blood available. The controls were transported to the irradiation facility with the experimental samples and held inside a polystyrene block to simulate exposure. Staff carrying out the irradiations also processed and scored the samples and were not blind to the radiation doses used.

### 2.3. Gamma H2AX Assay for Initial and Residual Radiation-Induced Foci

Following irradiation, the separated lymphocyte samples were incubated at 37 °C in a 5% CO_2_ humidified atmosphere to simulate in vivo repair. The 0.5 Gy samples were held for 0.5 h and the 4 Gy specimens for 24 h, to assess the initial and residual radiation-induced foci, respectively. Samples were then processed for the assessment of gamma-H2AX foci using a standard technique [22]. In brief, the lymphocytes were spotted onto adhesive slides, fixed with formaldehyde (Polysciences Inc., Warrington, PA, USA), permeabilised with Triton X (Sigma-Aldrich, Dorset, UK), blocked with bovine serum albumin (Fisher Scientific, Loughborough, UK) and immunostained for gamma-H2AX and 53BP1 using fluorophore-conjugated secondary antibodies (AlexaFluor 488 goat anti-mouse/AlexaFluor 555 goat anti-rabbit, Invitrogen, Paisley, UK). Fifty cells per sample were scored manually by one person, (with the exception of 8 donors), for gamma-H2AX and 53BP1 foci, using a Nikon Optiphot 2 fluorescence microscope equipped with separate filters to visualise 4′,6-diamidino-2-phenylindole (DAPI), fluorescein isothio-cyanate (FITC) and Texas Red. Only gamma-H2AX foci co-localised with 53BP1 were scored, as this reduces the possibility of erroneously scoring fluorescent antibody aggregates as foci [23].

### 2.4. Dicentric and Micronucleus Assays

For consistency with the standard protocol for the construction of calibration curves for biological dosimetry, whole blood samples were held for 2 h following irradiation at 37 °C, to allow for repair [3]. Whole blood was mixed with MEM for the DCA and RPMI medium (Sigma-Aldrich, Dorset, UK) for the MNA. MEM was used for the DCA for consistency with the protocol used to construct the X-ray dose–response curve used in this study [24], while RPMI has been the medium of choice in a previous study using an automated micronucleus analysis system [25]. The medium was supplemented with 10% FBS, 1% phytohaemagglutinin (Invitrogen, Paisley, UK), 100 units/mL penicillin plus 100 µg/mL streptomycin and 2 mM L-glutamine. In addition, 5-bromo-2-deoxyuridine and Colcemid (both from Sigma-Aldrich, Dorset, UK) were added to the DCA cultures at final concentrations of 10 and 0.04 µg/mL, respectively. All samples were cultured at 37 °C in a 5% CO_2_ humidified atmosphere. At 70 h, metaphases were harvested for the DCA by a standard hypotonic treatment in 0.075 M potassium chloride for 7 min at 37 °C followed by three changes of 3:1 methanol:acetic acid fixative. For the MN assay, the cell cultures had cytochalasin B (Sigma) added at 24 h, giving a final concentration of 6 μg/mL to block cytokinesis. The cells were harvested after a total of 72 h in culture by treatment with 0.075 M potassium chloride pre-cooled to 4 °C followed by fixation in methanol:acetic acid (3:1) with 1% formaldehyde. There followed two further changes of 3:1 methanol:acetic acid fixative. Fixed cells were dropped onto clean microscope slides, air dried and stained with 5% Giemsa (DCA) or 200 ng/mL DAPI in antifade mounting media (MNA). DAPI was used for the MNA instead of Giemsa as the automated detection software requires fluorescent images. The culture, fixation and staining procedures followed standard protocols recommended by the International Atomic Energy Agency [3]; although some MNA preparations were washed in phosphate buffered saline solution prior to staining to try and improve the quality of the stained preparations. Fifty metaphases per donor were scored manually for chromosome aberrations for the DCA, by 3 scorers. Dose estimates, based on the number of dicentrics per cell, were calculated using Dose Estimate_v5.1 [26] and dose–response curve coefficients C = 0.0005 ± 0.0005, α = 0.046 ± 0.005, β = 0.065 ± 0.003 [24]. A minimum of 500 binucleated cells (BN) per patient were scored automatically for the MNA on the Metafer slide scanning platform (Metasystems, Altlussheim, Germany), using the MSearch/MNScore software [27]. The data were recorded as fully automated scoring and with a cut-off for cells containing >4 micronuclei (MN). An estimate of the nuclear division index (NDI) was also calculated [3] for each sample.

### 2.5. DCA Calibration Curve Construction

The construction of the DCA calibration curve has been fully described by Lloyd et al. [24]. In brief, whole blood from a healthy male donor was exposed at 37 °C to 250 kV X-rays at a dose rate of 1.0 Gy/min. Eleven doses were used in total, ranging from 0.05 to 8.0 Gy. Following irradiation, the samples were held for 2 h at 37 °C and the cultures set up as described above, but without the addition of Colcemid at the start of the culture process. Samples were cultured at 37 °C for 48 h, with Colcemid added at 45 h to give a final concentration of 0.2 µg/mL. After 48 h, the cultures were fixed as described in Section 2.4 above for the DCA. The chromosome preparations were stained with orcein and scored for unstable aberrations. A later study [28] found similar yield coefficients, demonstrating that the data were not confounded by the presence of large numbers of second division cells despite using orcein stain.

### 2.6. Statistical Methods

Kolmogorov–Smirnov normality testing was carried out to assess the relevance of normal statistical analysis techniques. General linear model analysis of variance (GLM ANOVA) was then carried out, with post-hoc testing using Tukey’s pairwise comparisons between factors, using Minitab^®^ 17. For DCA, the factors tested were the scorer (3 individuals, pairwise comparisons); blood collection date (random variable); blood collection time (random variable); sample origin (4 hospitals) on dicentrics/cell, excess acentrics/cell and total aberrations/cell. Regression models were also applied for the aberration endpoints over time, to investigate an apparent variation in response. For the MNA, the model considered the influence of blood collection date, blood collection time and staining technique (washed or not washed prior to DAPI staining) on MN/cell (no cut off); MN/cell (cut off > 4 MN) and NDI. Data from a subset of 239 samples were used to investigate if the length of time-fixed samples had been stored in the freezer had an effect on the MNA endpoints. To ensure consistency between the time of making slides and scoring, this subset was used as the samples were all scored within 3 days of making slides regardless of storage time in the freezer. For the FCA, the scorer, stainer (2 individuals), blood collection date and time were assessed for the number of foci per cell at 24 h following 4 Gy or 0.5 h following 0.5 Gy. In addition, correlations between the endpoints were assessed using pairwise correlation analysis, to assess whether any single assay can be used as a homologue for the other endpoints. Finally, the Anderson–Darling test was used to investigate the distribution of the dose estimates from the DCA and then chi-squared testing, with Yates correction, was applied to compare each of the measured doses with the true dose.

## 3. Results

**Figure 1:**
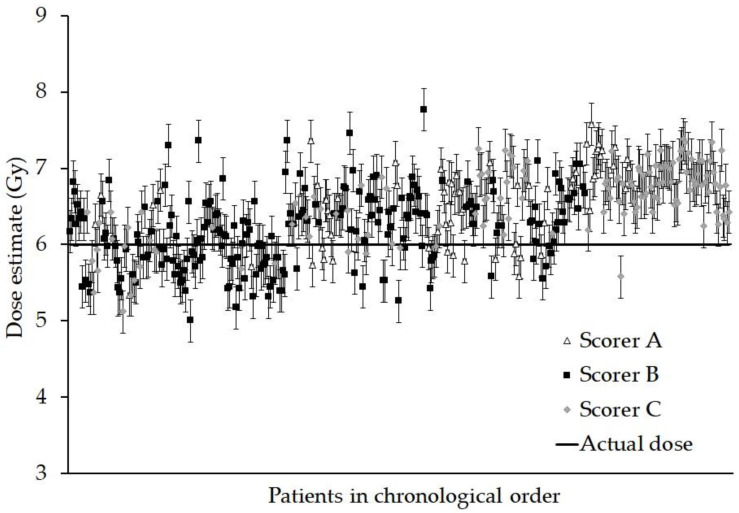
Graph showing the dose estimate and standard errors for each person. The data are presented in chronological order, (samples received over a continuous 18 month period) and for each scorer who analysed the sample. Every sample was given an ex vivo X-ray dose of 6 Gy.

Normality testing was carried out on the control and irradiated data and there was no evidence for significant deviation from normality for the majority of the DCA and foci endpoints. For the DCA, in Figure 1, the dose estimate and standard errors have been plotted for each patient in chronological order.

The Anderson–Darling test revealed a slight departure from normality in the distribution of the measured doses, chiefly due to a few outliers at each end of the scale, thus the chi-squared testing with Yates correction was applied to compare each of the measured doses with the true dose of 6 Gy. However, the results revealed that no one data point was significantly different from 6 Gy (all *p* > 0.6) and as a whole, the summed Yates’s chi-squared test suggests that the data are not significantly different from the true dose (*p* > 0.999). 

**Figure 2:**
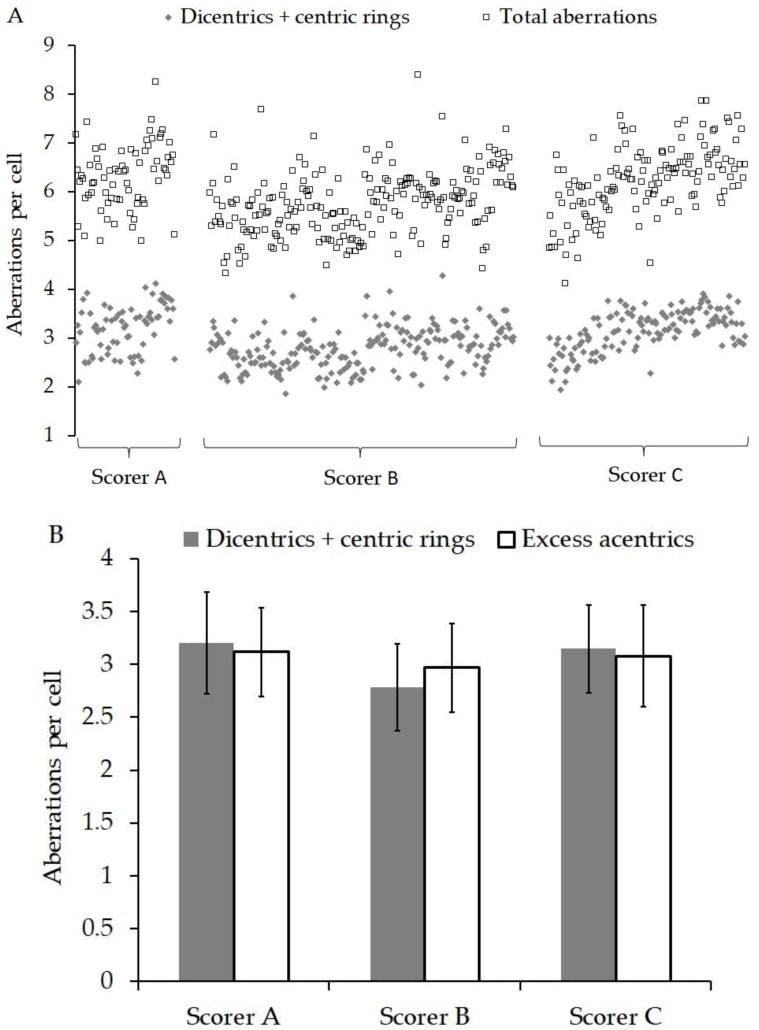
(**A**) Shows the yield of total aberrations (dicentrics + centric rings + excess acentrics) and dicentrics + centric rings found in each patient and grouped by scorer A, B or C. Within each group, the patients are arranged in chronological order. (**B**) shows the average yield of dicentrics + centric rings and excess acentrics analysed by scorers A, B and C. The errors bars represent the standard deviation.

Figure 2A shows the yield of total aberrations (dicentrics + centric rings + excess acentrics) and dicentrics + centric rings found in each patient and grouped by scorer A, B or C. Within each group, the patients are arranged in chronological order. Figure 2B shows the average yield of dicentrics + centric rings and excess acentrics analysed by scorers A, B and C. The GLM ANOVA indicated that the scorer was a significant factor for total aberrations/cell (*p* = 0.008), but not for excess acentrics/cell (*p* = 0.163) or dicentrics/cell (*p* = 0.146). The sampling time of day and the location from which it was sent had no significant influence on the aberration yields (*p* ≥ 0.656). However, the date of the blood sampling was significant for all the endpoints (*p* ≤ 0.005) but was independent of scorer interaction.

**Figure 3:**
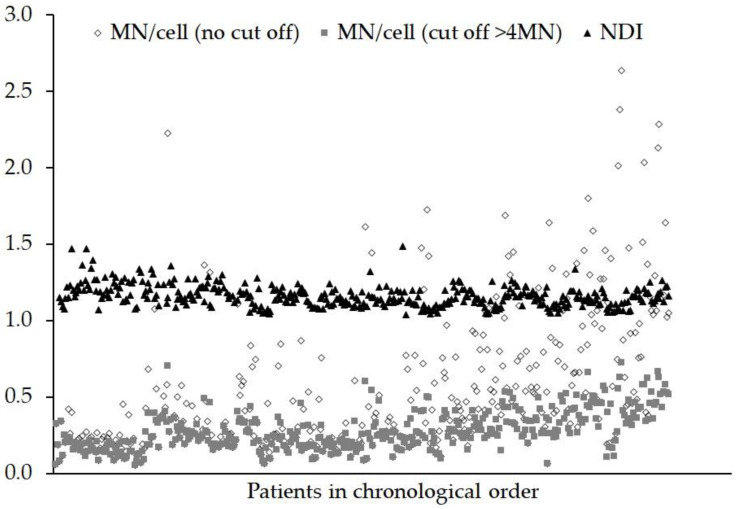
Graph showing the frequency of micronuclei (MN) observed and the nuclear division index (NDI) for each person after an ex vivo X-ray dose of 2 Gy. The data are presented in chronological order (samples received over a continuous 18 month period). Objects in bi-nucleated cells containing >4 MN tend to be artefacts or debris and can be eliminated using a cut off.

Shown in Figure 3 are the results of the MNA for MN/cell (no cut off), MN/cell (cut of >4 MN) and NDI for each patient, in chronological order. In common with the DCA, the date of the blood sampling was significant for all three MN endpoints (all *p* < 0.001). The sampling time of day was not significant for MN (cut off >4 MN) and NDI, (*p* = 0.247 and 0.867 respectively) and only showed a borderline significance for MN (no cut off), *p* = 0.049. The staining technique did not have a significant influence on the MN/cell when no cut off was used (*p* = 0.273), but for a MN/cell with a cut off >4 MN and for NDI, the techniques produced different results (*p* = 0.049 and 0.019, respectively). The length of time for which the fixed sample was stored in the freezer (0 to 58 weeks) had a significant effect on the MN/cell (no cut off) (*p* = 0.06), MN/cell (cut off > 4 MN) (*p* = 0.033) and NDI (*p* = 0.010).

**Figure 4:**
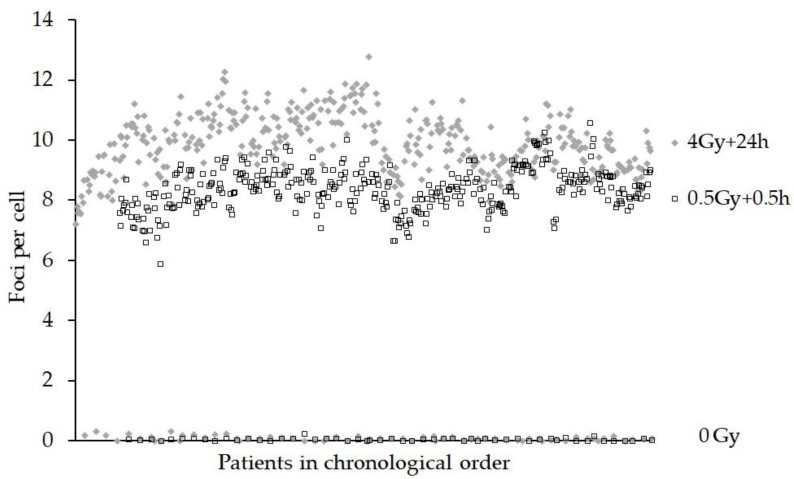
Graph showing the foci assay (FCA) results as gamma-H2AX foci per cell for 0 Gy, 0.5 Gy + 0.5 h and 4 Gy + 24 h ex vivo incubation at 37 °C samples. The data are presented in chronological order, (samples received over a continuous 18 month period).

Figure 4 shows the foci yields for each patient in chronological order for the 0 Gy, 0.5 Gy + 0.5 h and the 4 Gy + 24 h samples. ANOVA revealed that the effect of the scorer was not significant for the actual number of foci/cell (4 Gy + 24 h), *p* = 0.789, but only one batch of eight samples was scored by another person. In addition, the sampling time of day was not significant for 0.5 Gy + 0.5 h (*p* = 0.455) or 4 Gy + 24 h (*p* = 0.241). The effect of the person staining the samples was shown to be significant (*p* < 0.001), although all samples were stained by one person except for one batch of eight 0.5 Gy + 0.5 h samples. As with the DCA and MNA, the date of the blood sampling was significant (*p* = 0.001) for the FCA.

Cross endpoint correlations were carried out on dicentrics/cell, total aberrations/cell, MN/cell (no cut off), MN/cell (cut of >4 MN), foci/cell (0.5 Gy + 0.5 h) and foci/cell (4 Gy + 24 h). Most of the endpoints showed significant cross correlation, (*p* ≤ 0.050). No significant correlation occurred between foci/cell (4 Gy + 24 h) and dicentrics/cell (*p* = 0.531), total aberrations/cell (*p* = 0.179) or excess acentrics/cell (*p* = 0.153). In addition, the foci/cell (0.5 Gy + 0.5 h) were not significantly correlated with excess acentrics/cell (*p* = 0.660). MN/cell (cut of >4 MN) showed no significant correlation with excess acentrics/cell (*p* = 0.090). However, MN (no cut off) was significantly inversely correlated with NDI *p* < 0.001, but there was no significant correlation with MN (cut off >4 MN) and NDI *p* = 0.29.

## 4. Discussion

This paper looks at the logistics of carrying out the laboratory work and sample analysis for a large health effects study. A comparison of the cytogenetic and DNA damage data with the clinical scores of radiosensitivity will be the subject of a separate paper.

In this study, it was necessary for samples to be sent in batches, with a maximum of eight samples per batch. A total of 57 batches of samples were sent over an 18 month period. Only one batch of samples failed to arrive the next day and had to be repeated. In the present study, samples were only sent and received within the same country. Previous experience has shown that sending samples within the EU has been straight forward and that most samples arrive within 24 h [29,30]. Standard sample shipment outside of the EU, however, has been shown to be problematic, with delays as a result of country-specific import regulations that had to be overcome by using a specialised and expensive courier service [30].

The DCA was the most reliable assay with >99% of samples successfully completed, followed by the FCA (96%) and MNA (93%). Failures were caused by low cell numbers (all assays) or poor lymphocyte separation and bad staining (FCA). As a percentage of the total cost of the study, which includes reagents and staff costs (GBP 180,000), the MNA was the least expensive assay at 9% (GBP 16,000), followed by the FCA at 11% (GBP 20,000 for two time points), while the DCA was the most costly at nearly 25% (GBP 44,000). In part, this was due to the amount of effort required per sample. This was calculated to be 30, 50 and 120 min for the FCA, MNA and DCA, respectively. The use of automated scoring helped to keep the cost of the MNA low. If reagent costs alone are considered, the FCA is the most expensive and the DCA the least costly. Remarkably, the cost for the DCA, MNA and FCA together (45%) was lower than that for patient selection, recruitment, sampling and shipment in this study (55%).

All the patients had been historically exposed to ionizing radiation as treatment for either breast or prostate cancer [18,19]. The DCA and MNA assays used in this study measure unstable aberrations, for example, lymphocyte precursors carrying dicentrics or acentrics impose a hinderance to successful cell division and are eliminated over time. Dicentric yield therefore reduces with a half time of about 3 years [3], which is consistent with dicentric yields in the range of 0.005–0.070 per cell observed in patient baseline samples. In healthy non-exposed volunteers, background dicentric yields range from 0 to 0.002 per cell [31] and the excess aberrations in the patients’ baseline samples were considered to be very low compared to those induced by the 6 and 2 Gy ex vivo irradiation. The situation is even less critical for the FCA, which as a surrogate marker for radiation-induced DNA double-strand breaks shows a bi-exponential loss (fast and slow) of foci with time due to repair. Half times for the fast and slow repair have been shown to be 1.6 and 38 h, respectively [23].

As Figure 1 shows, there was a tendency for individual dose estimates to be above the 6 Gy actual dose (73%), although statistical testing suggested no single data point was significantly different from the true dose. One possible reason may be the slight variation in techniques between the application of the assay and the construction of the calibration curve, where blood was cultured for 48 h and Colcemid added at 45 h in the standard manner [24]. In the present study, a culture time of 70 h was used with Colcemid added at the start, albeit at a lower concentration. It is known that more heavily damaged cells reach metaphase later than cells containing fewer aberrations [32,33]. Despite using a lower concentration of Colcemid, the cells reaching metaphase sooner may have become more condensed and less easy to score and so have been more likely to be rejected by the scorer.

As described in the materials and methods, the scorers were not blinded to the radiation doses in this experiment. The main reason for this was the fact that doses were chosen on the basis of the indications in the literature that they could be useful for the prediction of normal tissue radiosensitivity, and in order to further investigate and compare the specific responses of the assays in this large scale scenario, rather than solely to test the ability of the assays to estimate the doses. Three scorers were involved in the analysis of the DCA. Scorer A had more than 30 years of experience using the DCA, while scorers B and C had 6 months and 2 years, respectively. Within the laboratory, all new staff underwent a training programme that compared intra-laboratory performance in dicentric yields and dose estimates. In addition, inter-laboratory comparisons were tested with images from collaborative projects, e.g., [34,35,36]. Figure 2 shows that on average scorer B scored lower than A and C, but using ANOVA, this was only significant for total aberrations. The DCA data appear to indicate that all aberration endpoints increased over time and the significant difference between scorers was related to the progression of time. This can be explained as changes in staff during the study resulted in scorer B analysing more samples at the start than at the end of the project.

In common with the DCA, the yields of MN show an upward trend with the progression of time; although this cannot be related to inter-scorer difference. One possible factor influencing MN yields was the time the fixed sample were stored in the freezer in 3:1 methanol:acetic acid. Time constraints, such as staff and microscope availability during the project resulted in the fixed cells from the MNA samples being stored in a freezer at −20 °C for anything from 0 to 58 weeks before the slides were made, stained and analysed. Most of the later samples were stored at −20 °C for less time than the earlier samples and ANOVA indicated the time spent in the freezer had a significant effect on all the MNA endpoints (*p* ≤ 0.033). Variable results in the automated MNA have also been reported by Depuydt et al. [37] with cells stored in fixative at 4 °C and −20 °C. One possibility is that the cells stored in fixative for long periods shrink or do not spread out well when dropped onto a slide. One of the parameters used by the MN classifier to detect bi-nucleated (BN) cells is based on size, hence different sub-sets of BN cells could be selected. Alternatively, fewer MN may be detected as a result of overlap with the main nuclei. When using automated analysis, all MNA samples should be stored and slides prepared in the same way as those used to train the MN classifier and produce dose–response curves.

For normal unexposed lymphocytes, the expected NDI values tend to be in the range from 1.30 to 2.20 [38]. Here, as shown in Figure 3, the range of NDI values was 1.05 to 1.50. These lower values probably reflect the fact that cells were delayed in their cell cycle after exposure to a 2 Gy genotoxic insult and that only the automated score of mono- and bi-nucleate cells were used to calculate the NDI. Figure 3 also shows that the range of values for the MN/cell (no cut off) is much wider than the MN/cell (cut off >4 MN), 0.06–2.64 and 0.06–0.73, respectively, especially towards the latter part of the study. Previous work [25], suggests that many of the objects in BN cells automatically classified as containing more than four MN tend to be artefacts or debris. In addition, the cross-correlation analysis showed no correlation with NDI for MN (cut off >4 MN), suggesting that when using automated scoring, cut off could be important.

A number of factors may influence the kinetics of foci formation/loss and small changes to reagents could affect staining quality [2,39]. Indeed, fluctuations in foci numbers were observed when new batches of gamma-H2AX antibody were used and may explain, or at least in part, the upward pattern of foci per cell that seems to repeat itself approximately every 5 months (see Figure 4). The GLM ANOVA showed the person staining the samples had a significant effect *p* < 0.001. All the samples were stained by one experienced person, except for one batch that was processed by a new less experienced member of staff. Batch normalisation, or the calculation of ratios for foci present at 24 vs. 0.5 h could be used to limit these effects when correlating this endpoint with, for example, clinical radiosensitivity.

## 5. Conclusions

Completing such a large scale study has provided some important points to consider for future studies. The experience of staff carrying out the assays is critical and laboratories involved in multi-partner studies must be very confident in their training/inter-comparison programmes. Moreover, if several laboratories undertake a joint study, the harmonisation of protocols and scoring would be essential. The cross endpoint correlation analysis of the present study showed that initial DNA damage, as measured by the FCA (0.5 Gy + 0.5 h), was significantly correlated with dicentrics/cell, that were the result of the mis-repair of double-strand breaks, but residual damage (FCA – 4 Gy + 24 h) was not. Therefore, it is important to choose assays carefully as they do not all measure the same thing and a multi-parametric approach may be more useful, as developed in the EU MULTIBIODOSE project [40]. A multi-centre approach to such studies can also increase the amount of soring that could be performed and ensure that samples can be analysed promptly and not stored for long periods of time, as this seems to be important for the automated MNA. The RENEB network already undertakes training and harmonisation activities to strengthen radiation exposure assessment for a number of biological dosimetry assays [16] and is ideally placed to carry out large health effect studies.
